# Primary Congenital Hallux Varus: A Step-Cut Surgical Approach

**DOI:** 10.7759/cureus.28075

**Published:** 2022-08-16

**Authors:** Panagiotis V Samelis, Panagiotis Kolovos, Stefania Nikolaou, Vasileios P Samelis, Nikolaos G Markeas

**Affiliations:** 1 Orthopaedics and Trauma, Children’s General Hospital Panagiotis & Aglaia Kyriakou, Athens, GRC; 2 Orthopaedics, Orthopaedic Research and Education Center, Attikon University Hospital, Athens, GRC; 3 Orthopaedics, Children’s General Hospital Panagiotis & Aglaia Kyriakou, Athens, GRC; 4 Microsurgery, Apostolos Pavlos Trauma Hospital, Athens, GRC; 5 School of Medicine, Department of Anatomy, European University of Cyprus, Nicosia, CYP; 6 Orthopaedics, Athens Children's Hospital, Athens, GRC

**Keywords:** hallux, varus, congenital, deformity, foot

## Abstract

Hallux varus is a rare deformity of the forefoot, which is characterized by medial deviation of the proximal phalanx of the great toe at the metatarsophalangeal joint. It is usually acquired, secondary to failed hallux valgus surgery, trauma, neurologic or rheumatologic disease. Rarely, this deformity may be congenital, either isolated, or in the context of various underlying congenital malformations of the foot, such as poly-syndactyly or longitudinal epiphyseal bracket, or part of generalized skeletal malformations. We present a case of bilateral congenital hallux varus with concomitant short first metatarsal in a three-year-old girl. A step-cut soft-tissue surgical procedure to achieve proper alignment of the medial ray of the foot is described.

## Introduction

Hallux varus is termed the medial deviation of the great toe at the metatarsophalangeal joint [1-3]. It should not be confused with the hallux adductus, which is accompanied by metatarsus primus varus [2]. The deformity may be congenital or acquired. Most cases are acquired, due to muscle imbalance in the first metatarsophalangeal joint [4-6]. Hallux varus is usually the result of failed hallux valgus surgery when both the adductor hallucis and the lateral head of the flexor hallucis brevis are released or due to excess medial eminence removal [3,4,6]. Other causes of acquired hallux varus are resection of the fibular sesamoid [4,7], congenital fibular sesamoid absence [6,8], or traumatic dislocation of the first metatarsophalangeal joint [4,6]. Rheumatoid disease, due to rupture of the lateral conjoint tendon of the great toe [8] and muscle imbalance due to neurologic disease may predispose to acquired hallux varus as well [6,8,9]. On the other hand, congenital hallux varus (CHV) is quite rare [6,10].

According to its etiology, CHV is divided into the primary type and secondary type [9,11,12]. In the primary type, no obvious underlying pathology is manifested. Janis et al. attributed the deformity to an intrinsic overactive abductor hallucis muscle (AbH) [9]. The secondary type, also termed the associated type of CHV, is observed in the context of skeletal malformations, such as great toe poly-syndactyly [1,2,13,14] or longitudinal epiphyseal bracket (LEB). The latter is usually located at the medial side of the first metatarsal and secondarily at the proximal phalanx of the toe (delta phalanx) [9,14]. A third type has been described, the tertiary type of CHV, in which hallux varus is associated with serious skeletal abnormalities, such as diastrophic dwarfism [11,13]. A short first metatarsal bone frequently accompanies the deformity [1,11,12]. A distinct fibrous band, which spans the base of the proximal phalanx and the base of the first metatarsal has been described [1,13]. This band is suggested to be a non-differentiated duplicate metatarsal [1,13]. The fibrous band may continue to an abnormally inserted tibialis anterior. Thus, tibialis anterior contraction may aggravate the deformity [13]. An autosomal dominant hereditary pattern has been supported, especially in the polydactylic - associated type of CHV [13], however, most cases of CHV seem to be non-hereditary [2].

CHV is not only a cosmetic problem but, most importantly, it affects the growing child’s ability to walk and play [1,10,13]. CHV deteriorates with growth, especially in the case of an underlying LEB [14]. To ensure normal sensory and motor development of the child, treatment of CHV should be completed by the end of the first year of life [12].

The treatment of a three-year-old girl with bilateral primary CHV is described.

## Case presentation

A term-born, third-born, three-year-old girl with bilateral CHV presented to the outpatient department. Mental and physical development were normal. The patient had no history of traumatic, neurologic or other pathology, nor had she a family history or clinical or radiologic evidence of skeletal disease. According to her parents, the great toe deformity was evident since birth. The deformity impaired with age. The patient did not receive any treatment until this age. Shoe wearing was quite uncomfortable and normal gait was impossible.

**Figure 1:**
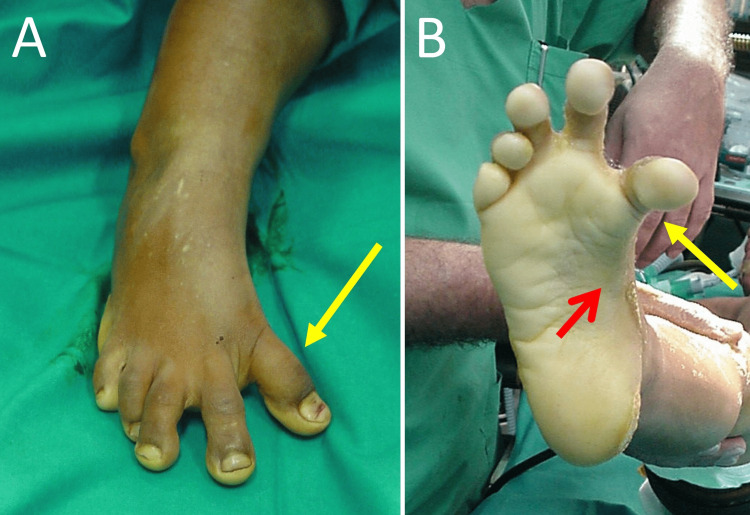
Hallux varus of the right foot in a 3-year-old girl with bilateral congenital hallux varus A. Dorsal view of the right foot: hallux varus (yellow arrow), B. Plantar view of the right foot: hallux varus (yellow arrow), inversion of the foot and pes cavus (red arrow)

On clinical examination, marked varus of the great toe at the first metatarsophalangeal joint was evident. The hallux could not be realigned to the first metatarsal with manual pressure from the examiner, indicating a fixed deformity. Pes cavus, inversion of the foot and mild clawing of all toes were also evident. These deformities did not correct with manipulations from the examiner as well (Figure 1).

**Figure 2:**
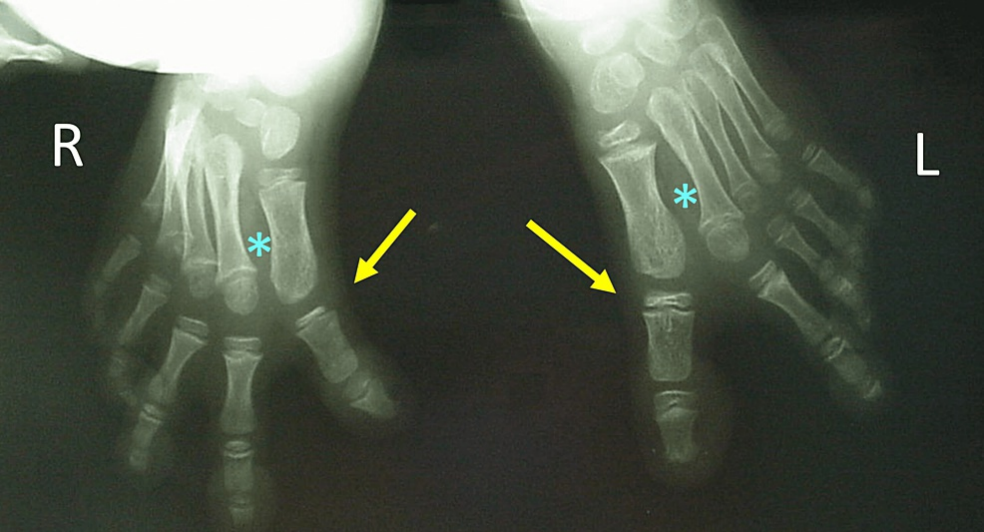
Anteroposterior x-ray view in a 3-year-old girl with bilateral congenital hallux varus The level of the deformity is at the first metatarsophalangeal joint (yellow arrows). The intermetatarsal angle is normal (asterisk)

Except of a short first metatarsal, the radiologic examination did not reveal sings of associated type of CHV, such as a LEB of the first metatarsal of the proximal phalanx of the great toe, poly-syndactyly, or first metatarsal duplication. The first intermetatarsal angle was normal (Figure 2).

Staged treatment of the entire deformity, starting with the right foot, was decided. A dorsomedial curved incision between the base of the proximal phalanx of the great toe and the medial malleolus was performed. The AbH, along with dense connective tissue on the medial side of the proximal phalanx and the first metatarsal was resected. The medial cortex of the first metatarsal was exposed. There was no intraoperative evidence of a LEB on the medial side of the first metatarsal. Capsulotomy of the first metatarsophalangeal joint followed. The extensor hallucis longus tendon was freed from the surrounding connective tissue. Syndactylization at the first web space completed the correction of the great toe deformity.

**Figure 3:**
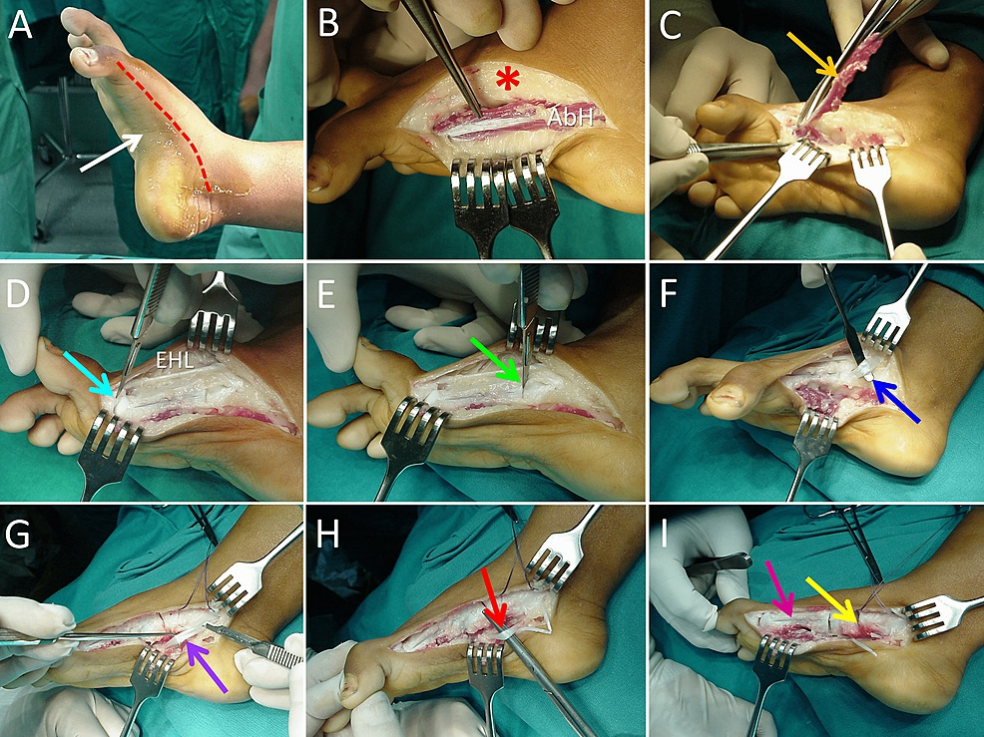
The procedure A: Skin incision (red line), pes cavus (white arrow), B: Dorsomedial exposure of the medial aspect of the foot (asterisk). The abductor hallucis muscle (AbH) is exposed, C: Resection of the AbH (orange arrow), D: Capsulotomy of the 1st metatarsophalangeal joint (blue arrow), the entire length of the tendon of the extensor hallucis longus (EHL) was freed from the surrounding fibrous tissue, E: Capsulotomy of the 1st metatarsocuneiform joint (green arrow), F: Transfer of the tibialis anterior insertion to the 3rd metatarsal base (deep blue arrow), G: Z-lengthening of tibialis posterior (purple arrow), H: Z-lengthening of flexor digitorum longus and flexor hallucis longus (red arrow), I: Capsulotomy of the medial cuneiformnavicular joint and the talonavicular joint (yellow arrow). The medial cortex of the first metatarsal is exposed (pink arrow).

To correct pes cavus and foot inversion, capsulotomy of the first cuneometatarsal, first naviculocuneiform and talonavicular joints followed. Dense connective tissue over the insertion of the tibialis anterior was removed. The tibialis anterior tendon was transferred to the third metatarsal base. Z-type elongation of the tendon of tibialis posterior followed. The operation was completed with flexor digitorum longus and flexor hallucis longus muscle z-lengthening (Figure 3).

**Figure 4:**
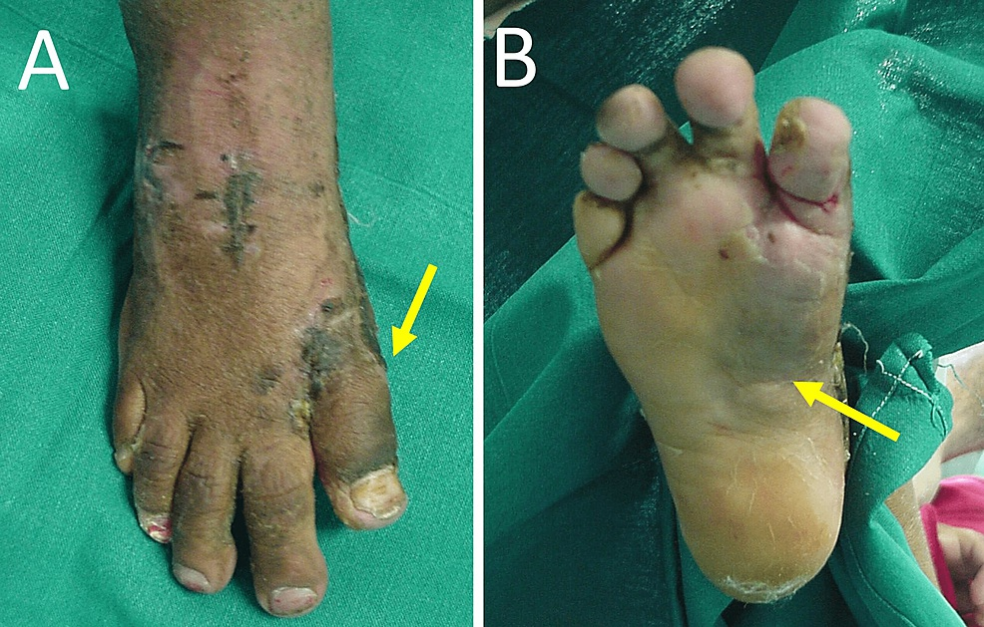
The patient six weeks after surgery. The plaster is removed A. Complete correction of the varus deformity of the great toe (arrow). B. The medial longitudinal arch and the version of the foot have been restored (arrow)

After completion of the soft-tissue procedure, the alignment of the medial ray of the foot was restored, without additional manual pressure from the surgeon. Transfixation of the metatarsophalangeal joint or osseous interventions, such as osteotomy or joint fusions, were deemed unnecessary. A gutter splint was placed on the medial side of the foot for six weeks to secure medial row alignment until soft tissue healing was complete (Figure 4). Progressive weight bearing was recommended.

After six months, mild, painless limitation of motion of the first metatarsophalangeal joint was manifested. The patient was walking without obvious discomfort from the operated foot. No recurrence of the deformity, neither signs of skin hyperkeratosis, due to excess pressure from the footwear were evident. First metatarsal lengthening will be decided in future if necessary to improve gait. Surgical treatment of the left foot was planned.

## Discussion

CHV is quite rare [3,10,13,14]. Most reports are case reports or very small case series. The majority of cases are of the associated type [14]. Serial casting may be attempted in mild primary CHV, however, surgical treatment is recommended by most authors. Surgical treatment of CHV has been described since more than 80 years ago [1]. Due to the rarity of the deformity and the complexity of associated skeletal malformations, various procedures have been described, and none can be recommended over the other [12,14].

McElvenny described the surgical procedure on a patient with associated type CHV in the background of a duplicated great toe [1]. The fibrous band on the medial side of the proximal phalanx and the first metatarsal were removed. Medial release of the first metatarsophalangeal joint by means of medial capsulotomy followed. Subsequently, the lateral and dorsal capsule was reinforced by means of a tendon transfer of the extensor hallucis brevis through the first metatarsal head and then around the extensor expansion of the great toe. Syndactylization of the first web space completed the correction of the deformity. The foot was protected in a cast for one month [1].

Farmer described eight cases of CHV, most of the associated type [2]. He incised all soft tissue structures (skin, AbH, connective tissue, capsule) at the medial aspect of the metatarsophalangeal joint. No tendon transfers were implemented. Syndactylization of the first web space followed. The skin of the broad first web space was used as a dorsal or plantar rotation flap to cover the medial skin defect after the reduction of the great toe on the first metatarsal head [2]. A cast was applied for three weeks to maintain the correction of the forefoot [2]. Resection of the AbH has also been described [14].

These basic soft tissue techniques have been used by most authors to treat CHV, isolated or in combination with osseous procedures. In general, soft tissue procedures are performed in cases with a normal first metatarsal [14]. Supernumerary bones are excised if present [14]. Osteotomies are reserved for cases with LEB or in very severe deformities, which cannot be addressed only with soft tissue procedures [14]. Fusion of the first metatarsophalangeal joint is rarely used, usually in neglected cases with a nonfunctional or painful first metatarsophalangeal joint [10]. Amputation of the hallux has been applied as a secondary procedure after poor results of the index surgery [10]. First metatarsal lengthening for cosmetic reasons is not recommended, because of the risk of abnormal loading of the foot during the gait cycle [10].

We present a surgical procedure to address all aspects of a congenitally deformed foot, in a child with primary CHV. We started with resection of the AbH. Medial capsulotomy of the first metatarsophalangeal joint followed. The surgical intervention was extended step-by-step to the articulations and tendons of the medial aspect of the foot to address pes cavus and fixed foot inversion. We did not find a distinct fibrous band between the proximal phalanx and the base of the first metatarsal, as described by McElvenny [1]. Our procedure resembles in part the surgical treatment of idiopathic congenital talipes equinovarus because the deformity of the presented case was mainly the result of abnormally developed connective tissue and of contractions of the capsule and the tendons of the medial part of the foot. Soft tissue release stopped when the deformity was corrected without further manipulation from the surgeon.

## Conclusions

A rare case of bilateral primary congenital hallux varus is presented. Stepwise resection and loosening of contracted muscles, tendons, and joint capsules of the medial part of the foot is a safe procedure to restore the forefoot anatomy. Osseous procedures, such as metatarsal osteotomy or fusion of the first metatarsophalangeal joint should be reserved for the older patient if adequate correction cannot be obtained only by soft tissue procedures. First metatarsal lengthening for cosmetic reasons carries the risk of creating symptoms in a previously asymptomatic foot.
